# Mammalian cortical voltage imaging using genetically encoded voltage indicators: a review honoring professor Amiram Grinvald

**DOI:** 10.1117/1.NPh.4.3.031214

**Published:** 2017-05-04

**Authors:** Chenchen Song, Samuel Barnes, Thomas Knöpfel

**Affiliations:** a Imperial College London, Laboratory for Neuronal Circuit Dynamics, London, United Kingdom; b Imperial College London, Division of Brain Sciences, London, United Kingdom; c Institute of Biomedical Engineering, Imperial College London, Centre for Neurotechnology, South Kensington, London, United Kingdom

**Keywords:** optical imaging, genetically encoded voltage indicators, voltage-sensitive fluorescent proteins, mesoscopic imaging, cortical dynamics

## Abstract

The pioneering work of Amiram Grinvald established voltage-sensitive dye imaging (VSDI) in the mammalian cortex in the 1980s and inspired decades of cortical voltage imaging and the associated technological developments. The recent conception and development of genetically encoded voltage indicators (GEVIs) overcome many of the limitations of classical VSDI, and open experimental approaches that provide accruing support for orchestrated neuronal circuit dynamics of spatially distributed neuronal circuit underlying behaviors. We will review recent achievements using GEVIs to optically monitor the cortical activity in mammalian brains *in vivo* and provide a perspective for potential future directions.

## 1 Introduction

The brain, and in particular the human brain, is the most functionally complex mammalian organ. Fundamental to the function of the brain is short-lasting (in the range of milliseconds) changes of the plasma membrane potential of neurons. It has also long been recognized that approaches that enable the monitoring of these electrical activities are required to elucidate the brain’s function in the processing and storage of information and the generation of behavior. Indeed, a multitude of available techniques were developed to achieve this goal, ranging from high-temporal resolution but with low-spatial coverage techniques, such as single-cell electrophysiology, to low-temporal resolution but with high-spatial coverage techniques, such as functional magnetic resonance imaging. Despite the functional values and clinical benefits of these techniques, their main limitation is the lack of combined temporal and spatial resolution and coverage. Understanding electrical activity at the single cell level is a fundamental goal of neuroscience, but large area monitoring of electrical activity at high-spatiotemporal resolution and coverage is also important for understanding the many emergent properties of neural network-level processing.

The early revolutionary work of Amiram Grinvald and colleagues took the important first step toward tackling the challenge of developing high-spatiotemporal resolution in the intact brain by establishing voltage-sensitive dye imaging (VSDI) of cortical electrical activities many decades ago. This approach relies on the topical application of voltage-sensitive dyes (VSDs) through a craniotomy to monitor the cortical activity.^1^ The more recently developed genetically encoded voltage indicators (GEVIs) report, such as VSDs, membrane potential activity in the form of changes in optically detected fluorescence intensities while overcoming the methodological limitations associated with craniotomies and dye delivery.

GEVIs improve upon classic VSDs in several additional aspects: (i) they allow targeted expression in genetically identified cell classes with highly reproducible expression patterns across population, (ii) they provide reliable recordings from these specific cell populations over prolonged periods and across multiple sessions, and (iii) they permit noninvasive optical cortical monitoring in thin-cranium species such as mice. In addition, GEVIs provide direct real-time monitoring of neuronal electrical activity to uncover additional information including subthreshold activity and hyperpolarizing events, which cannot be detected using other genetically encoded optical indicators that report neuronal activity via surrogate signals (e.g., changes in calcium concentrations). As discussed in Antic et al.,^2^ classic VSDs may still have advantages when applied in *ex vivo* preparation or *in vivo* for questions in which sub-millisecond time resolution is critical, for example, the investigation of plasticity involving changes in the timing of neurotransmission.^3^

In our lab we have generated a series of GEVIs termed voltage-sensitive fluorescent proteins (VSFPs) that are based on the voltage-sensor domain of *Ciona intestinalis* voltage-sensing phosphatase.^4^^–^^6^ Based on this original design concept and several additional discoveries and contributions of others working in the field, there is now a growing palette of GEVIs.^7^ The molecular and biophysical properties of currently available GEVIs have been covered by several recent review articles.^2^^,^^6^^–^^8^ In the context of the present review, we would like to highlight two technical considerations: (i) although many new GEVIs show promising properties when tested in cultured cells, only a few GEVIs have been rigorously validated *in vivo*. In our hands, the absolute amplitudes of optical GEVI signals are typically much smaller *in vivo* than *in vitro* (as is known for classical VSDs this is caused by a larger background signal), but typically comparable or better than those of widely used VSDs^9^ and (ii) many new and most-sensitive GEVIs are used with a single greenish fluorescence emission wavelength. As known for VSDs used before the development of “blue” [red and near infrared (NIR) fluorescence-emitting] VSDs, this feature causes complications in *in vivo* experiments due to the strong hemoglobin absorption in this wavelength range.^10^ GEVIs with two anticorrelated emission wavelengths [i.e., Förster resonance energy transfer (FRET)-based sensors] are, therefore, strongly preferable.

Here, we focus on the application of VSFPs *in vivo* in the mammalian system, with particular emphasis on transgenic animals expressing the VSFP Butterfly family^11^^–^^13^ of FRET-based GEVIs, to achieve targeted cortical monitoring of electrical activity of identified neuronal classes in head-fixed anesthetized and awake mice through noninvasive cranial window implants.^11^^,^^14^

## 2 Mesoscopic Monitoring of Cortical Activity

Understanding how neuronal activity is transformed into complex behaviors, such as perception, sensory–motor integration, and eventual cognition and consciousness, is at the core of contemporary systems and circuit neuroscience. Cortical neuronal computation is distributed across large-scale networks over substantial cortical space, thus to optically observe circuit operations at the population level, i.e., mesoscopically, it is necessary to decipher information processing and to achieve functional mapping in the mammalian cortex.

The pioneering work of Grinvald and colleagues^14^ on optical monitoring of cortical activity using classic VSDs has inspired decades of mesoscopic voltage imaging to understand population-level neuronal computations, yet VSDI also has obvious methodological limitations. Cortical tissue comprises a heterogeneous neuronal population which VSDs label indiscriminately. Cortical VSDI is dominated by activity from the cortical glutamatergic populations because of their greater number, while contributing to diverse functions with no lesser importance are the GABAergic interneuron population which comprises only about 20% of the cortical cell population. This methodological challenge has been overcome by GEVI voltage imaging via genetic targeting of GEVIs to specific cell classes. This selective activity monitoring of specific populations is a crucial step toward an accurate description and reconstruction of dynamic cortical network interactions.

**Fig. 1:**
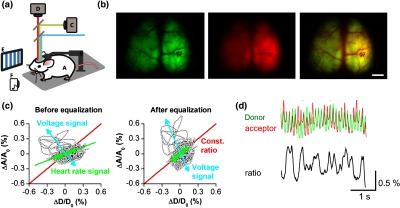
Acquiring and preprocessing of epifluorescence signals from FRET-based ratiometric GEVIs. (a) A GEVI-expressing transgenic mouse [A] is trained to be head-fixed [B] after cranial window implant. Cortical activity is imaged under a dual-channel imaging set-up using two CCD cameras to monitor the fluorescence intensity changes of the FRET donor [C] and acceptor [D] signals simultaneously. Sensory stimulations (shown are visual gratings [E] and auditory tones [F] as examples) can be applied during optical imaging. (b) Left: mCitrine (donor) image; middle: mKate (acceptor) image; right: overlay of registered images. Scale bar = 3 mm. (c) The signals derived from the two cameras reflect both voltage and hemodynamic responses. Heartbeat-related fluctuations in excitation and emission light absorption are observed in both donor and acceptor channel and are corrected by equalization of their amplitude before calculation of the acceptor/donor ratio. Left: before equalization; right: after equalization. (d) The FRET pair of fluorescent proteins anticorrelate in fluorescence intensity to reflect fluctuations in membrane potential during stimulus-free spontaneous activity to produce a ratiometric optical read-out. (b)–(d) adapted with permission from Akemann et al.^11^

GEVI transgenic mice have achieved cell class-specific expression of the VSFP Butterflies, a ratiometric GEVI family that uses modulation of FRET between two fluorescent proteins as the voltage reporting mechanism. GEVI-based cortical voltage imaging is achieved through transcranial monitoring and can cover large cortical areas (often simultaneously across both hemispheres) in head-fixed anesthetized and awake animals [Fig. 1(a)]. The fluorescence intensities of the two fluorescent proteins are simultaneously monitored using two cameras after a dichroic mirror that splits emitted light into the donor and the acceptor spectral bands. Structural images show the prominent cortical vascular signatures that are used for preprocessing registration, and as landmarks for multisession registration and relocation of regions of interest [Fig. 1(b)].

Voltage indicator signals (both VSD and GEVIs) acquired with light at the visible range of wavelengths from the mammalian brain *in vivo* contain, in addition to the voltage signal, signals that are ascribed as hemodynamic response. The hemodynamic response has two principal components: (i) the pulsating blood flow causes rhythmic blood volume fluctuations at the heart beat frequency^15^ causing changes in absorbance in the optical path of excitation and emission light. Increase in blood volume results in decrease of indicator fluorescence and (ii) neuronal activity is associated with changes in hemoglobin oxygenation. The sign of this effect is wavelength dependent.

The dual emission feature of FRET-based GEVIs provides an opportunity not only to correct for hemodynamic signal components (separation of hemodynamic responses from the voltage signals) [Figs. 1(c) and 1(d)] but also to simultaneously monitor voltage and hemodynamic responses.^11^^,^^14^

## 3 Cortex-Wide Intrinsic Population Activity

A large body of work by Amiram Grinvald and colleagues has demonstrated that VSDI of mammalian cortex can resolve the dynamics of synchronized activities which occur in the absence of sensory input.^16^^–^^20^ This nonevoked activity is referred to as “spontaneous,” “ongoing” or “internal activity,” and is observed both in the developing and adult brains, in line with classical and more recent work using electrophysiological and calcium imaging techniques.^21^^–^^24^

Combined VSD optical imaging and electrophysiology have demonstrated that spontaneous synaptically-driven fluctuations of membrane potential of single neurons are often coherent time-locked events rather than independent processes,^25^ indicating that such on-going cortical activity represents not just neuronal noise but reflects local and brain-wide orchestration of activities.^16^^,^^26^ Anatomically adjacent neurons can belong to different functional assemblies, yet the neuronal spontaneous firing is shown to be tightly linked to the cortical networks to which they belong.^17^ This is further supported by recent evidence suggesting a resemblance between spatial patterns of on-going activity motifs and activation maps obtained with sensory stimulation.^27^

**Fig. 2:**
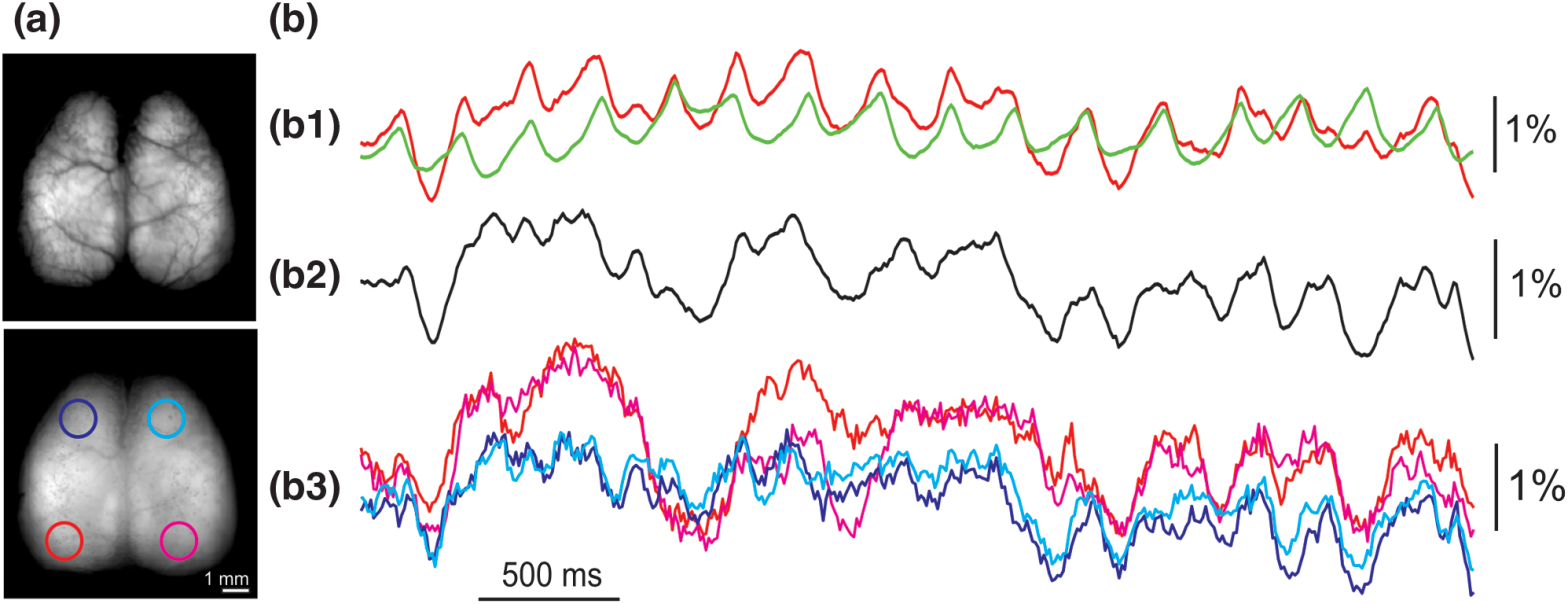
Intrinsic activity in the absence of sensory stimuli monitored in transgenic mice expressing VSFP Butterfly 1.2 in layer II/III pyramidal neurons. (a) Dorsal view over both cortical hemispheres of a mouse with a chronically implanted transcranial window, captured through the FRET donor (upper) and acceptor (lower) channels. Four ROIs are outlined as: (i) left motor cortex (navy), (ii) right motor cortex (cyan), (iii) left visual cortex (red), and (iv) right visual cortex (pink). (b) Intrinsic activity imaged across both hemispheres in the absence of sensory stimulation under light sedation. Upper: individual donor (green) and acceptor (red) fluorescence signals averaged across both hemispheres; middle: ratiometric voltage signal across both hemispheres; lower: ratiometric voltage signal of intrinsic activity across the four ROIs [left motor cortex (navy), right motor cortex (cyan), left visual cortex (red), and right visual cortex (pink)] as outlined in A lower, showing isotopic cortical activity traversing across large distance in both hemispheres. These data were collected as described in Akemann et al.^11^ and Carandini et al.^14^

More recently, GEVI-based imaging confirmed many of the aforementioned observations.^11^^,^^28^^,^^29^ Cortex-wide mesoscopic voltage monitoring using the GEVI VSFP Butterfly 1.2 revealed spontaneous waves of activity of mouse layer two of three cortical pyramidal neuronal populations (Fig. 2). These waves traverse across the entire field of view, that is the dorsal aspect of the mouse cortex, and are observable across different anesthetic brain states, during slow wave sleep and during resting wakefulness.^11^^,^^29^^–^^31^ Interestingly, clustering analysis of the spatiotemporal patterns of these activity waves reveals a small set of recurrent motifs. Across different brain states, these motifs are conserved but with systematic changes in their dynamics.^29^

**Fig. 3:**
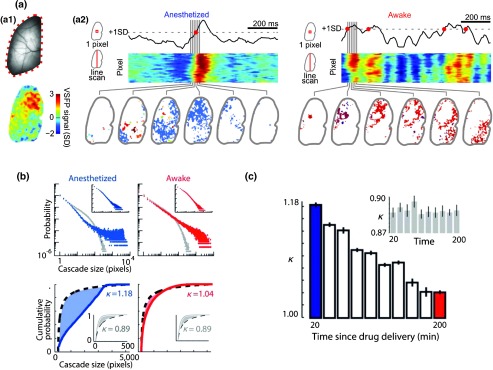
Spontaneously occurring cascades of population depolarizations show brain state-dependent emergence of criticality. (a) Voltage imaging in a head-fixed mouse through a dorsal transcranial window (one hemisphere shown). Upper: baseline fluorescence image. Lower: map of VSFP Butterfly 1.2 voltage signal for a 20-ms snapshot. (a1) Data from an anesthetized mouse (left) and a mouse in resting wakefulness state (right). Upper: voltage imaging traces from one pixel (33×33  μm cortical area). A pixel is considered to be active at times when the signal crosses a threshold from below (red circles). Middle: voltage signals from a vertical line of pixels reveal spatiotemporally contiguous activity; color code as in a1. Lower: consecutive snapshots of cascades of active pixels during a 120-ms period. Each cascade has been labeled with a unique color. (b) Cascade size probability distributions approach power-law form during the recovery from anesthesia. Upper: probability distribution of cascade sizes, in number of active pixels, in anesthetized (left, blue), and awake resting (right, red) states. Each data point represents the probability Pr(z) of observing a cascade of size z. Very large cascades were prevalent in the anesthetized state. The probability distribution for resting awake-state mouse is close to a power law. Gray points show probability distributions after randomized circular permutation of single pixel traces relative to each other, a control with no cross-pixel correlations. Data presented are for the 10 image sequences recorded over a 20-min period in anesthetized states (20 to 40 min) and in resting states (200 to 220 min) in one mouse. Insets show cluster size distributions for the anesthetized and resting states. Lower: cumulative probability distributions for anesthetized (left, blue) and resting (right, red) states. Deviations from a reference power law with exponent −1.5 (black dashed lines) are quantified by κ, which measures the gap (shaded area) between the measured distribution and the reference distribution. The closer κ is to 1, the closer the distribution is to the reference. Shuffled control distributions for both states showed similar deviation from the reference distribution, with κ<1 (insets, gray). (c) Values of κ calculated for image sequences recorded over 20-min periods, and shuffled data (inset, gray). Error bars represent SEM of 10 consecutive sequences. Adapted with permission from Scott et al.^30^

Experimental characterization of VSDI- and GEVI-based imaging of intrinsic activity patterns has been complemented by theoretical frameworks for neuronal circuit dynamics.^30^^–^^32^ The theory of criticality is one such potential framework that suggests that cortical networks self organize to operate at the dynamic boundary of local interactions and large-scale cross-cortical synchronizations where the rules of interactions are scale invariant.^33^^,^^34^ Recent GEVI-based optical imaging of unperturbed cortex has shown that the features of spontaneous clusters of up-states (imaged as slow waves of population depolarizations) are in fact brain state dependent: criticality emerges with awakening, in which the dominance of long-range (cross cortical) interactions characteristic for slow wave sleep and anesthesia decreases^30^ (Fig. 3). Analysis of GEVI optical imaging data in the framework of information theory supported the postulation that information capacity within the cortical network increases with the emergence of criticality.^31^^,^^35^

## 4 Cortical Representation of Sensory Information

The physiological mechanisms underlying cortical sensory processing have long been established from using single cell-level electrophysiology,^36^^–^^39^ but how sensory stimuli are processed involving top-down influences and long-range connections required monitoring representations across larger cortical areas. Cortical VSDI, as inspired by the early work of Grinvald and colleagues,^40^ has contributed much to our understanding of sensory neurophysiology at the level of local cortical networks but also, perhaps even more importantly, at the level of cortex-wide interarea connectivities. With the aforementioned stochastic ongoing spontaneous activity averaged out over repeated presentation of the sensory stimulus, VSDI has generated maps of sensory representation in the visual,^41^^–^^45^ auditory,^46^^–^^48^ somatosensory cortices,^15^^,^^49^^–^^51^ and the olfactory bulb,^52^ and provided basic insights as to the cortical processing of neuronal information.

Following its initial application in invertebrates, amphibians, cats, and monkeys, the use of VSDI in cortical sensory processing was extended to rodents, in which earlier work using VSDI had established that peripheral stimulations of whiskers elicit a “field of activity” extending over in the somatosensory cortical columns.^50^ Subsequent work confirmed this observation, and at an improved spatiotemporal resolution, resolved the initial activity to be confined to a single column.^49^ The physiological extent of this activity spread, as mapped by VSDI, is correlated to the stimulation strength, weaker whisker deflections elicit depolarization confined to a single barrel column, intermediate deflections cause depolarization to spread into adjacent barrel columns preferentially asymmetrically along whisker rows, whereas large deflections generate a depolarizing event spreading over the barrel field. Later work also confirmed the triphasic response of this elicited activity observed by Kleinfeld and Delaney,^50^ a rapid initial depolarization phase, followed by hyperpolarization, and ending with a long-lasting rebound depolarization.^51^^,^^53^ The stimulus-sensitive hyperpolarizing phase appeared in the form of an asymmetrical ring that is more distant from the epicentre, providing spatiotemporal insights into the mechanism by which the cortex processes sensory information, such as lateral inhibition^54^^,^^55^ and gain normalization.^56^^–^^58^

Sensory perception at the cortex-wide (interarea) level relies on the dynamic function of long-range connections,^59^^–^^61^ and VSDI contributed much to connectivity mapping and the characterization of cortical activity propagation *in vivo*.^62^^–^^64^ Long-range cortical interactions have been shown using VSDI in experiments in which brief whisker deflection elicits an initial depolarizing response in the contralateral primary somatosensory (S1) barrel cortex that generates secondary localized activity in “satellite regions,”^50^ such as the primary motor cortex. Secondary activity is also observed in the ipsilateral cortical regions, resulting in eventual apparent bilaterally symmetrical depolarizing activity spreading to other cortical regions with complex spatiotemporal dynamics.^65^

Connections that are involved in higher-order sensory processing extend beyond the supragranular layers into deeper cortical layers and subcortical structures (e.g., thalamus). Long-distance functional connectomics have been facilitated by combining VSDI of superficial cortical layers with photostimulation of deeper cortical layers using optogenetic actuators expressed in restricted cell classes.^66^^,^^67^ This approach capitalizes on the superior target selectivity of optogenetic stimulation compared with electrical microstimulation, but does not resolve the heterogeneous nature of VSDI signals and the restricted monitoring to superficial cortical layers, in which all cell types of the superficial layers and the apical dendrites of the deeper layers are indiscriminately represented in the optical voltage signal.

**Fig. 4:**
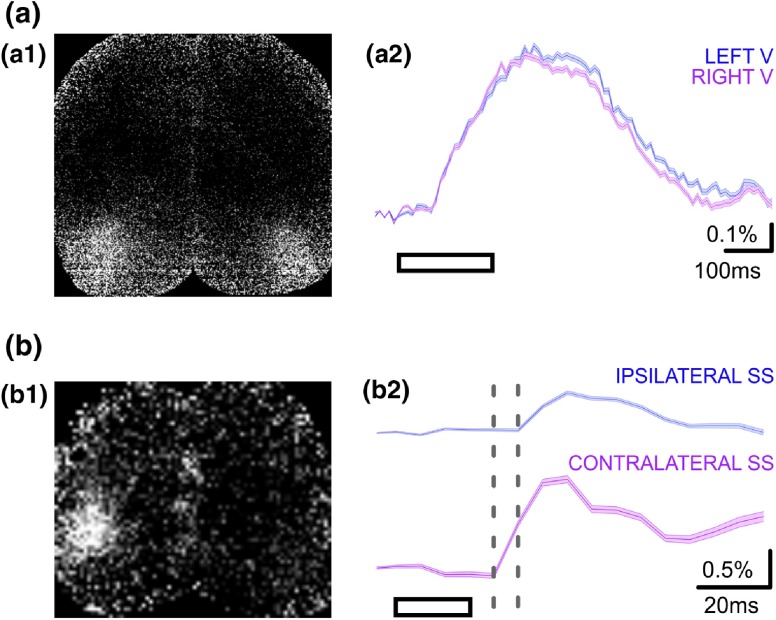
Cortical representation of sensory stimuli in slightly sedated mice. (a) Cortical responses to bilateral visual stimulation (200-ms horizontal moving grating) in a transgenic mouse expressing VSFP Butterfly 1.2 in layer II/III pyramidal population. (a1): ΔR/R snapshot showing peak depolarizing response in both visual cortices following binocular sensory stimulation. (a2): Ratiometric depolarizing responses from the left and right visual cortex ROIs, averaged across 40 trials (±SEM). (b) Cortical response to right whisker stimulation (20 ms air puff to whole trimmed whisker field) in a transgenic mouse expressing chimeric VSFP Butterfly in pyramidal neurons across all cortical layers. (b1): ΔR/R snapshot showing peak depolarizing response in the contralateral somatosensory cortex following sensory stimulation. (b2): Ratiometric depolarizing response from the contralateral and ipsilateral somatosensory ROIs, averaged across 50 trials (±SEM). Fast depolarizing response is initially observed in the contralateral ROI, and subsequently in the ipsilateral ROI with a minor delay. Gray dotted lines demark time period represented by the snapshot shown in b1. Note that the ipsilateral response appears only in the subsequent frame. Note that the different response timescales in (a) and (b). Mice are sedated. These data were collected as described in Akemann et al.^11^ and Carandini et al.^14^

GEVI-based voltage imaging therefore serves as a perfect opportunity for monitoring neuronal responses in both a cell class-specific (e.g., pyramidal neurons versus GABAergic interneurons) and anatomically defined (e.g., dendrites of supragranular layer pyramids versus apical dendrites from supgranular pyramidal cells) manner, to achieve precise reconstruction of the functional dynamics of sensory processing. Functional mapping of sensory processing in layer II/III pyramidal populations using ratiometric GEVI have been realized initially using VSFP2,^28^ and subsequently using VSFP Butterfly 1.2.^11^^,^^14^ This was later also achieved via transgenic strategies^12^^,^^13^ for enhanced stringent control over the optical signal origin. In transgenic mice expressing VSFP Butterfly 1.2 in layer II/III pyramidal neurons, the binocular presentation of horizontal drifting gratings elicited isotopic depolarizing activity initiating from the primary visual cortices in both hemispheres [Fig. 4(a)] which is temporally comparable with previous observations using electrophysiological measures.^68^

Somatosensory stimulation of head-fixed transgenic animals expressing the GEVI chimeric VSFP Butterfly in all cortical pyramidal neurons have also produced rapid depolarizing homeotopic response in the somatosensory cortex [Fig. 4(b)]. This depolarizing response is observed both in the contralateral and the ipsilateral barrel cortices with the ipsilateral responses appearing after a small delay, similar to previous observations using VSDI.^65^ Expression of the same GEVI in all cortical GABAergic cell types allowed the selective monitoring of somatosensory processing in the interneuron populations only, providing insights into the distinctive excitatory and inhibitory components of dynamic neuronal circuits. Because the balance between excitation and inhibition in the neuronal circuits have major implications for sensory processing,^69^^–^^71^ this ability to discern the excitatory and inhibitory components of sensory processing also has profound implications for understanding normal neurophysiology^72^^,^^73^ as well as diseased states.^74^

## 5 2-Photon GEVI Voltage Imaging

Voltage maps obtained with classical VSDs in wide-field mode show, at best, blurred cellular morphologies due to out-of-focus light and light scattering. The optically reported membrane voltage *in vivo* in wide-field mode imaging experiments is a compound voltage reflecting contributions of stained membranes within a larger tissue volume delimited axially by the penetration length of visible light (<200  μm). Although a visualization of coarse compound activity often provides a useful representation of cortical dynamics by emphasizing the major modes of activity shared by a larger set of neurons, there are other questions requiring sharper resolution.

The foremost optical approach to improve spatial resolution and deliver unblurred optical sections of micrometer thickness up to larger tissue depth is 2-photon microscopy.^75^ However, 2-photon voltage imaging has been lagging as a monitoring method, mostly because of the low photon counts expected in the 2-photon image scan acquisition mode at fast frame rate unavoidably leading to high-photon shot noise.^76^ Nevertheless, *in vitro* studies recently demonstrated single-trial sensitivity for recording of action potentials in axonal terminal arbors,^77^ back-propagated action potentials in single spines,^78^ and spontaneous and evoked somatic potentials in neurons in acute brain slice^79^ using 2-photon imaging of VSD variants. On the other hand, available 2-photon imaging data of sensory-evoked activity in somatosensory and visual cortex remained poor in signal-to-noise requiring extensive trial averages (>100) to surpass the noise barrier.^76^^,^^80^ More recently, 2-photon voltage imaging using GEVIs was explored with promising conclusions.^80^^–^^82^

**Fig. 5:**
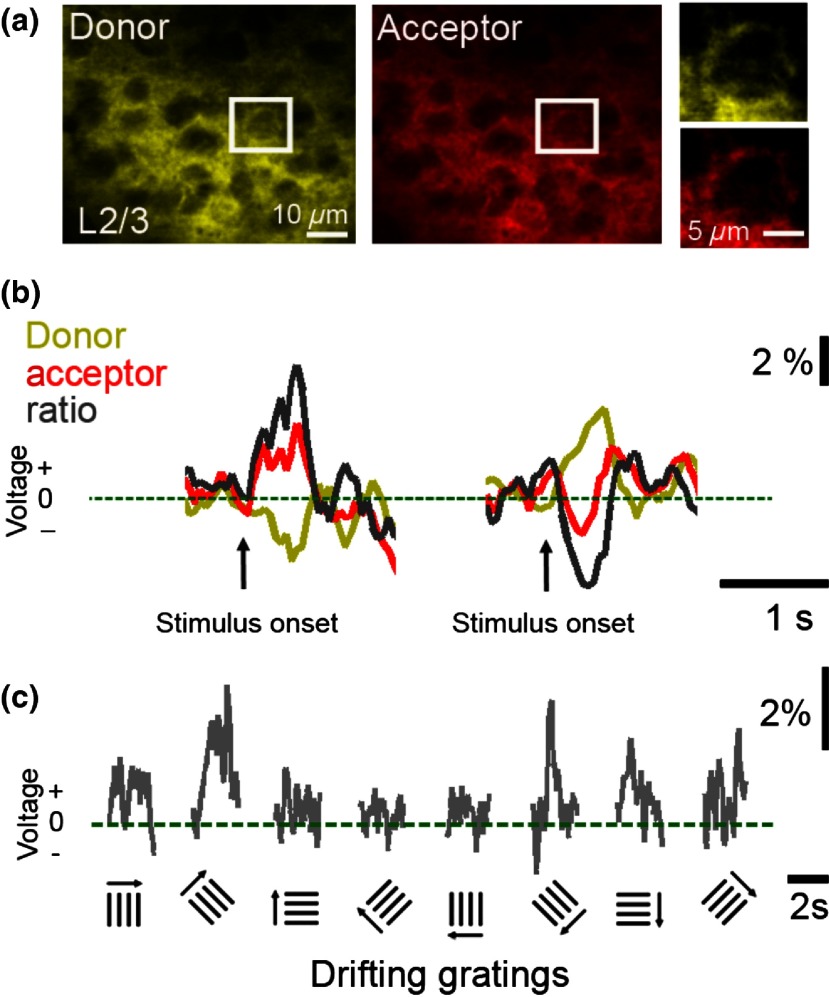
2-Photon *in vivo* voltage imaging of sensory processing under sedation. (a) Example of *in vivo* cortical regions from a transgenic mouse expressing chimeric VSFP Butterfly in all pyramidal neurons. A single layer II/III pyramidal neuron is outlined in the ROI. (b) Single-trial *in vivo* voltage responses to auditory stimuli from the pyramidal neuron in the identified ROI in (a) (right), where different auditory stimuli can elicit either depolarizing response (left) or hyperpolarizing response (right). (c) Example of average voltage responses to visual stimulation (drifting gratings of different orientations; number of trials = 10). These data are unpublished examples generated using the methods described in Akemann et al.^81^

We established the feasibility of 2-photon voltage imaging in the mouse cortex at single cell resolution using Butterfly GEVIs^81^ (Fig. 5). The optical voltage signals in these recordings have a dynamic range of 1% to 3%, about two to three times larger than in 1-photon recording (but 1 to 2 orders of magnitude smaller than typical calcium imaging signals). The increased signals observed with 2-photon imaging compared with 1-photon excitation modes can be explained by less nonresponsive background fluorescence in the 2-photon case. The dynamic range of 2-photon voltage signals may be small compared with those of calcium indicators but in addition to the modest signal size, cellular level voltage imaging is further complicated by the fact that most of the volume averaged optical voltage signals is generated by neuronal dendrites.^15^^,^^49^^,^^83^ This latter aspect could be resolved by the promising efforts in attaining optimum sparse indicator expression in individual neurons *in vivo*.

## 6 Future Directions

It has been pointed out that the first GEVI was published around the same time as the first genetically encoded calcium indicator (GECI), but it may be interesting to note that the first GEVIs did not function in mammalian cells, whereas the first GECIs did. Iterative improvement of GECIs resulted in more probes with greater sensitivity that are now widely used to monitor calcium transients in the live mammalian brain.^84^ It is reasonable to expect that the development of GEVIs will follow this path. Indeed, there are intense current efforts by several groups and consortia toward improved GEVIs and associated technologies, many of which are supported via the BRAIN initiative. But voltage imaging will remain more challenging and more difficult than the widely and successfully used calcium imaging approaches, be it only because the signals of interest are typically faster (voltage transients versus calcium transients). Beyond efforts to improve the effective sensitivity of GEVIs, current efforts focus on sparse targeting (to facilitate allocation of signals to individual neurons), subcellular targeting selectivity (soma dendritic versus axonal membranes to maximize the ratio between signal photons and noise photons) and indicator color variants that extend into the NIR. NIR GEVIs would be tremendously useful for combination with blue/greenish light-mediated optogenetic photostimulation and deep tissue imaging toward further delineating the functional causal mechanisms underlying *in vivo* neurophysiology.
